# GLUT4 Defects in Adipose Tissue Are Early Signs of Metabolic Alterations in *Alms1^GT/GT^*, a Mouse Model for Obesity and Insulin Resistance

**DOI:** 10.1371/journal.pone.0109540

**Published:** 2014-10-09

**Authors:** Francesca Favaretto, Gabriella Milan, Gayle B. Collin, Jan D. Marshall, Fabio Stasi, Pietro Maffei, Roberto Vettor, Jürgen K. Naggert

**Affiliations:** 1 Department of Medicine, University of Padua, Padua, Italy; 2 The Jackson Laboratory, Bar Harbor, Maine, United States of America; GDC, Germany

## Abstract

Dysregulation of signaling pathways in adipose tissue leading to insulin resistance can contribute to the development of obesity-related metabolic disorders. Alström Syndrome, a recessive ciliopathy, caused by mutations in *ALMS1*, is characterized by progressive metabolic alterations such as childhood obesity, hyperinsulinemia, and type 2 diabetes. Here we investigated the role of *Alms1* disruption in AT expansion and insulin responsiveness in a murine model for Alström Syndrome. A gene trap insertion in *Alms1* on the insulin sensitive C57BL6/Ei genetic background leads to early hyperinsulinemia and a progressive increase in body weight. At 6 weeks of age, before the onset of the metabolic disease, the mutant mice had enlarged fat depots with hypertrophic adipocytes, but without signs of inflammation. Expression of lipogenic enzymes was increased. Pre-adipocytes isolated from mutant animals demonstrated normal adipogenic differentiation but gave rise to mature adipocytes with reduced insulin-stimulated glucose uptake. Assessment of whole body glucose homeostasis revealed glucose intolerance. Insulin stimulation resulted in proper AKT phosphorylation in adipose tissue. However, the total amount of glucose transporter 4 (SLC4A2) and its translocation to the plasma membrane were reduced in mutant adipose depots compared to wildtype littermates. Alterations in insulin stimulated trafficking of glucose transporter 4 are an early sign of metabolic dysfunction in Alström mutant mice, providing a possible explanation for the reduced glucose uptake and the compensatory hyperinsulinemia. The metabolic signaling deficits either reside downstream or are independent of AKT activation and suggest a role for ALMS1 in GLUT4 trafficking. Alström mutant mice represent an interesting model for the development of metabolic disease in which adipose tissue with a reduced glucose uptake can expand by de novo lipogenesis to an obese state.

## Introduction

Increased prevalence of obesity and diabetes, often associated with reduced lifespan, is a worldwide problem in the human population. Obesity is a consequence of an imbalance between food intake and energy expenditure. Adipose tissue (AT) acts as an energy depot to maintain metabolic homeostasis, ensuring a rapid response to modifications of nutrient availability. Proper AT expandability is necessary to accommodate excess nutrients and to avoid peripheral lipotoxicity. Obesity is characterized by AT expansion through hyperplasia and/or hypertrophy [1] and by the presence of dysfunctional AT with fibrosis, altered angiogenesis and inflammation, and often associated with local and systemic insulin resistance (IR) [2].

It is generally thought [3], [4], [5] that hyperinsulinemia triggers the expansion of AT in the early phase of obesity and IR of muscle and adipose tissues appears later, suggesting that adipogenesis requires insulin-sensitive fat cells. However, patients with lipodystrophy exhibit high insulin levels but reduced AT depots [6]. This discordance suggests a complex regulation of AT insulin sensitivity, adipogenesis and IR.

In AT, insulin stimulates glucose entry by a specific carrier, the solute carrier family 2 (SLC2A4), also known as glucose transporter 4 (GLUT4), whose alterations have been related to local and systemic IR [7]. GLUT4 is able to dynamically cycle among the different subcellular compartments along microtubules and actin fibers. In the basal state, most of the transporters are located within specialized intracellular vesicles and organelles, including the trans-Golgi network (TGN), recycling endosomes (REs) and tubulo-vesicular structures. In response to insulin or contraction stimulations, most of the transporters are rapidly translocated to the plasma membrane (PM) where they take up extracellular glucose and are then recycled and stored until new stimulation occurs [8].

Alström syndrome [ALMS (MIM #203800)] is a rare autosomal recessive disease characterized by the progressive development of severe multiorgan pathology with metabolic alterations. ALMS patients exhibit early hyperinsulinemia and childhood obesity, that typically converts to a lower body lipodystrophic pattern [9]. The associations between the molecular deficits in ALMS and the development of obesity and IR have not been well characterized.

ALMS is caused by mutations in *ALMS1*, a ubiquitously expressed gene located on chromosome 2p13 [10], [11]. ALMS1 localizes to centrosomes and basal bodies of ciliated cells and has putative roles in cell cycle regulation [12], [13], ciliary function, cytoplasmic microtubular organization, intracellular and endosomal transport [14], [15], [16], [17], [18].

In the present work, we sought to elucidate the mechanisms of obesity and insulin resistance in ALMS using an *Alms1* genetrap mouse model. Previously, we reported that, in *Alms1^GT/GT^* mice, body weight and insulin levels begin to increase between 8 and 12 weeks of age [15]. Here, we demonstrate that disruption of *Alms1* induces alterations of adipocyte morphology and gene expression profile in 6 week old mice, before the onset of obesity and hyperinsulinemia. In *Alms1^GT/GT^* AT, we observed a reduction in total GLUT4 content, in insulin-stimulated GLUT4 translocation and glucose uptake without impairment of adipogenesis. Our findings suggest a direct role of ALMS1 in glucose homeostasis via the GLUT4 trafficking pathway.

## Materials and Methods

### Animal care and handling

*B6Ei.129S2-Alms1^Gt(XH152)Byg/Pjn^* (*Alms1^GT/GT^*) and littermate control (wild type, wt) male mice were genotyped as previously described [15]. Mice were fed a 5K54 diet with 4% fat (LabDiets, Richmond, IN, USA) *ad libitum* and provided unlimited access to water (HCl acidified, pH 2.8–3.2) in a temperature and humidity controlled setting with a 12 h light/dark cycle.

### Ethics statements

All procedures were approved by the Institutional Animal Care and Use Committee at The Jackson Laboratory (Permit Number: AUS#99089), according to the “Guide for the Care and Use of Laboratory Animals”; all efforts were made to minimize animal suffering.

### Biochemical measurements

Male mice were weighed and heparinized whole blood was collected at 4, 6, 8, 12, 16, 18, 20, 21 weeks of age from the orbital sinus, anesthetized with tetracaine drops (n = 8–15/group). Plasma chemistries [glucose, cholesterol, high density lipoproteins (HDL), low density lipoproteins (LDL), triglycerides, alanine aminotransferase (ALT), free fatty acids], were measured using a Beckman Coulter Synchron CX5 Delta chemistry analyzer (Beckman AU680, Brea, CA, USA). Plasma insulin and leptin levels were measured using specific ELISA kits (ALPCO Diagnostics, Salem, NH, USA & Crystal Chem Inc, Downers Grove, IL, USA). To calculate the homeostasis model assessment of insulin resistance (HOMA-IR) index, blood plasma was collected from mice (n = 4–5/group) after fasting for>6 hours. HOMA-IR was calculated as follows: (glucose (mg/ml) X insulin (µU/ml))/405 [19].

### Histological and immunohistochemical analysis

After CO_2_ euthanasia, inguinal subcutaneous (SAT) and epididymal visceral adipose tissues (VAT) were collected from 6 Alms1^GT/GT^ and 6 wild type (wt) male mice. Briefly, a median longitudinal incision was made (neck to pubis) on the mouse's ventral side, while repositioning the skin away from the body. SAT was extracted from 6 week old mice at the inguinal region anterior to the upper segment of the hind limbs (beneath the skin), as depicted by Lim *et al* [20]. Tissues were fixed in 4% paraformaldehyde, paraffin-embedded, and sectioned. Sections were de-paraffinized with xylene and dehydrated by a series of alcohol washes. For histology, tissues were stained with hematoxylin-eosin (H&E) and images taken using the ScanScope CS digital slide scanner and analyzed with Aperio software (Aperio Technologies, Vista, CA, USA). For immunohistochemical detection of F4/80 (Abcam; ab6640), the Ventana Discovery XT system (Ventana Medical Systems) was used per manufacturers protocol. Briefly, de-paraffinized tissues were incubated with primary antibody (1∶200) following a protease 1 digestion for 8 minutes. The Detection kit used was ChromoMap DAB, a multimer-based HRP kit (Ventana Medical Systems) using an anti-rat secondary antibody (OmniMap anti-Rt HRP, 760–4457) and DAB as the chromogen. Sections were counterstained with Mayer's hematoxylin and viewed with a Leica DMLB microscope. Digital images were captured using Q Imaging software (Surrey, British Columbia, Canada).

### Cell culture

#### Mouse pre-adipocyte primary cultures

SAT pools (5 *Alms1^GT/GT^* and 8 wt) were minced and digested in a collagenase type II solution (1 mg/ml) (Sigma-Aldrich, St. Louis, MO, USA), centrifuged at 350×g, and red blood cells were lysed using buffer (NH_4_Cl 1.545 M, KHCO_3_ 100 mM, EDTA 1.27 mM) as previously described [21]. Cells from the stromal vascular fraction (including pre-adipocytes) were seeded at a density of 2.5×10^5^ cell/cm^2^ in 24 well plates (CellStar, Kremsmuenster, Austria) in standard medium 10% FBS (SdM: DMEM F12 supplemented with 150 U/ml streptomycin, 200 U/ml penicillin, 2 mM glutamine, 1 mM HEPES (GIBCO, Invitrogen Ltd, Paisley, UK). When cells were confluent, the medium was replaced with adipogenic medium (AdM) containing 66 nM insulin, 100 nM dexamethasone, 1 nM T3, 0.25 mM IBMX, 10 µM rosiglitazone in SdM 5% FBS. IBMX and rosiglitazone were removed after 3 days of culture. After day 7, AdM was replaced with SdM and cells were grown for at least 2 days before further analysis.

### Assessment of *in vitro* glucose uptake

The 2-[1–3H] deoxyglucose (2-DG) uptake assay was performed as previously described [22] with minor modifications. Cells differentiated in 24-well plates were incubated for 2 h in serum-free medium and then stimulated in triplicate for 1 h at 37°C with insulin (0, 100 nM and 2 µM). The test was initiated by incubating for 15 minutes at 37°C in a solution of D-glucose (50 µM) and 2-DG (1.5 µCi/ml) (GE Healthcare, Waukesha, Wisconsin, USA). The test was terminated by two rapid washes in ice-cold PBS. Cells were solubilized with 0.1% Triton X-100/PBS and the radioactivity was measured using a β-counter (PerkinElmer, Waltham, MA, USA). An aliquot of each cell lysate was removed for total protein quantification with a Micro BCA Assay Kit (Thermo Fisher Scientific Inc., Waltham, MA, USA). The insulin-induced glucose uptake was normalized to total protein content and reported as percent increase with respect to the basal value.

### Oil-Red O staining and triglyceride quantification

Cells were fixed for 1 h in 10% formalin/PBS at 4°C and stained with Oil-Red O (Sigma-Aldrich) solution in 40% isopropanol for 15 min at room temperature (RT). After 3 washes with PBS, Oil-Red O staining (ORO) was extracted in isopropanol and the absorbance was measured spectrophotometrically at 518 nm (Beckman Coulter).

### RNA extraction and RT-Real time PCR

Total RNA was extracted using RNeasy Lipid or Mini Kits (QIAGEN) following the supplier's instructions. For each sample, 1 µg of RNA was treated with DNase Treatment & Removal Reagents (Ambion, Inc, Austin, TX, USA) and reverse-transcribed for 1 h at 37°C with 150 ng random hexamers, 0.5 mM dNTPs, 20 units of RNAs in Ribonuclease Inhibitor and 200 units of M-MLV RT (Promega, Madison, WI, USA). Oligonucleotide sequences and amplification conditions are reported in the Table S1. Real Time PCR was carried out with Platinum SYBR Green qPCR SuperMix-UDG (Invitrogen) on a DNA Engine Opticon 2 Continuous Fluorescence Detection System (MJ Research, MA, USA). Duplicate 5 ng cDNA samples were normalized by *Rn18s* (*18S rRNA*) content and reported as arbitrary units ratio.

### Preparation of subcellular fractions

Male mice were fasted for 4 h after the dark cycle and then treated with insulin (0.5 U/kg; n = 9) or saline (n = 9) by intraperitoneal injection. After 30 minutes, SAT was collected and immediately frozen in liquid N_2_. Cells from 2 wells of a 6 well-plate per experiment were serum-starved for 2 h and treated for 1 h at 37°C with (n = 3) or without (n = 3) 2 µM insulin. Subcellular fractions were prepared as previously described [23], with minor modifications. Briefly, the whole tissue pooled from 3 mice or cells were homogenized in ice-cold buffer A (20 mM Tris-HCl, 1 mM EDTA, 255 mM sucrose, pH 7.4) with EDTA-free protease inhibitor cocktail (Roche Molecular Biochemicals, Indianapolis, IN, USA). After lipid and debris removal, samples were centrifuged for 20 min at 19,000×g and the resulting supernatant, containing the microsomal membrane fraction, was stored at 4°C. The pellet, containing the PM-rich fraction, was re-suspended in 3 ml of buffer B (20 mM Tris-HCl, 1 mM EDTA, pH 7.4) with protease inhibitor and layered onto a 6 ml sucrose cushion (38% sucrose in Buffer B), and centrifuged at 100,000×g for 70 min. The PMs were collected at the interface of the sucrose cushion, re-suspended in buffer B and centrifuged at 40,000×g for 20 min. The initial supernatant was centrifuged for 20 min at 41,000×g, producing the high-density microsome fraction (endoplasmic reticulum compartment, HD) pellet. The supernatant recovered from the previous centrifugation was re-centrifuged at 180,000×g for 70 min, allowing collection of a low-density microsome fraction (REs compartment, LD). All pellets were resuspended in buffer A with protease inhibitors and samples were quantified using the Micro BCA Assay Kit.

### Western Blot Analysis

SAT and VAT from 6 *Alms1^GT/GT^* and 6 wt mice where homogenized in ice cold RIPA buffer (1% Np40, 0.5% sodium deoxycholate, 0.1% SDS) with PhosSTOP and/or EDTA-free protease inhibitor cocktail (Roche, Indianapolis IN, USA). Total protein content was quantified using a Coomassie (Bradford) Protein Assay Kit (Thermo Fisher Scientific Inc., Waltham, MA, USA). Thirty µg of total protein or 8 µg of purified subcellular fractions for each mouse were loaded onto 4–12% NuPAGE Novex Bis-Tris Gels (Invitrogen) for electrophoresis and subsequently transferred to a nitrocellulose membrane (Pall Corp., Pensacola, FL, USA). Membranes were blocked with BlottoA (5% milk, 0.1% Tween in TBS) for 1 hour at RT and then incubated with anti-GLUT4 antibody (1∶5000–7500 Abcam, Cambridge, UK), pAKT S473 (1∶1000 Cell Signaling, Danvers, MA, USA), AKT (1∶1000 Santa Cruz, Dallas, TX, USA), β actin (1∶2000, Sigma-Aldrich) and GAPDH (1∶1500, Sigma-Aldrich) overnight at 4°C. The membranes were washed in 1X TBS several times, and then incubated with horseradish peroxidase-conjugated secondary anti-rabbit antibody (1∶40000-Jackson ImmunoResearch, Europe Ltd, Suffolk, UK) for 1 hour at RT. After several washes in TBS, signal was detected using an ECL chemiluminescent detection system (GE Healthcare). Captured images were quantified using ImageJ software (http://rsb.info.nih.gov/ij/).

### Glucose and insulin tolerance tests

To examine the effects of *Alms1* disruption on whole body glucose homeostasis, 6 week old male mice were fasted for 6 hours followed by intraperitoneal injection of glucose (1 mg/g body weight; Sigma) or insulin (1 U/kg body weight; Eli Lilly, Indianapolis, IN, USA). Blood was collected via tail tips at timed intervals (0, 15, 30, 60, 120 min) following injection. Glucose levels were measured using a glucometer (Onetouch Ultra, Lifescan, Johnson & Johnson).

### Statistics

Results are presented as mean ±SEM or SD. Statistical significance was determined using unpaired Student's t (two-tailed) and Mann-Whitney tests. Differences were considered significant with P<0.05. Areas under the curve (AUC) were calculated using the “trapz” function with the R package “pracma” (Practical Numerical Math Functions).

## Results

### Studies *in vivo*: characterization of adipose tissue depots

#### Metabolic characterization of Alms1^GT/GT^ on the C57BL6/Ei genetic background

**Figure 1:**
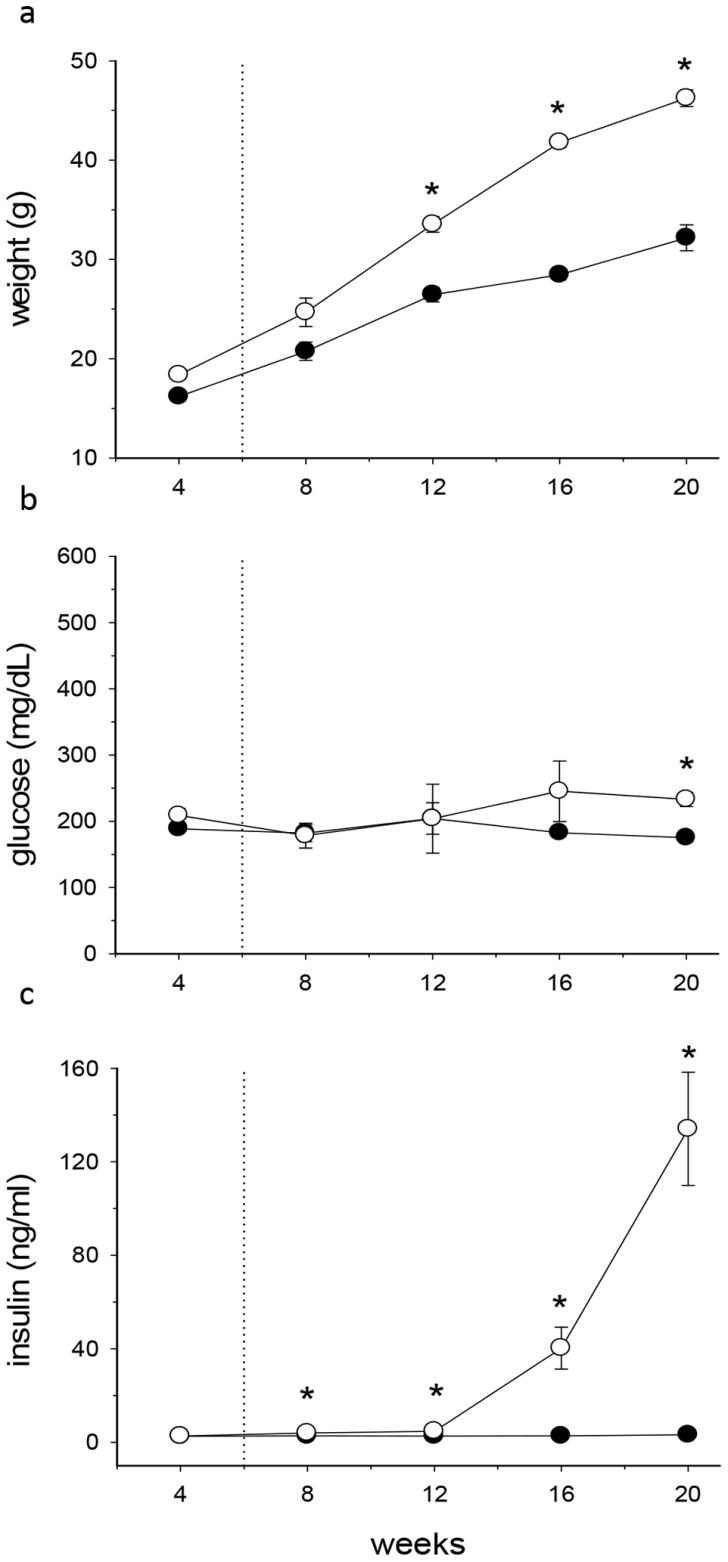
Metabolic parameters in wt (black symbols) and Alms1^GT/GT^ (white symbols) male mice. The body weight (a), plasma glucose (b) and insulin levels (c) were evaluated at 4 week intervals. Dotted lines denote the age (6 weeks) of mice characterized in the present study. Data are expressed as mean values ±SEM, n = 15. *p<0.01 wt *vs*. *Alms1^GT/GT^*.

**Figure 2:**
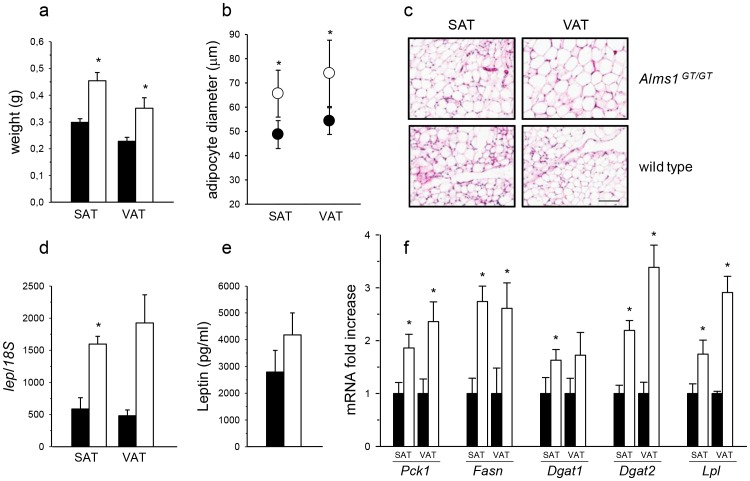
Adipose tissue characterization in 6 week-old Alms1^GT/GT^ mice. (a) Weight of subcutaneous (SAT) and visceral (VAT) tissues of wt (black bars) and *Alms1^GT/GT^* (white bars) mice. *p<0.01 wt *vs*. *Alms1^GT/GT^*. (b) Average of 100 adipocyte diameters in SAT and VAT of 6 *Alms1^GT/GT^* (white circles) and 6 wt (black circles) expressed as mean values ±SD. *p<0.01 wt *vs*. mutant animals. (c) A representative H&E staining of adipose tissue depots in *Alms1^GT/GT^* and wt mice. Scale bar = 100 µm. (d) Leptin expression in SAT and VAT of 6 wt (black bars) and 6 *Alms1^GT/GT^* (white bars) mice. Data are normalized to *Rn18s* (*18S*) content and reported as arbitrary unit mean ratio ±SEM. *p<0.05 wt *vs*. *Alms1^GT/GT^*. (e) Circulating plasma leptin in 12 wt (black bars) and 12 *Alms1^GT/GT^* (white bars) mice. Data are expressed as mean values ±SEM. (f) mRNA expression of several enzymes involved in different lipogenic pathways in SAT and VAT of 6 wt (black bars) and 6 *Alms1^GT/GT^* (white bars) mice (*Pck1*: Phosphoenolpyruvate carboxykinase 1, *Fasn*: Fatty acid synthase, *Dgat1*: Diacylglycerol acyltransferase 1, *Dgat2*: Diacylglycerol acyltransferase 2, *Lpl*: Lipoprotein lipase). Each transcript was normalized to *Rn18s* content. Results are reported as arbitrary unit mean ratio ±SEM and are expressed as fold change with respect to wt, arbitrarily set as 1 for each transcript. *p<0.05 wt *vs*. *Alms1^GT/GT^*.

*Alms1^GT/GT^* mice described by Collin et al. [15] harbor an insertion of a gene trap cassette in intron 13 of *Alms1*. On a mixed (C57BL/6J and 129P1/ReJ) genetic background, mice developed obesity, hyperinsulinemia, and hyperglycemia. Toye *et al* showed that C57BL6/J mice carry a deletion in the nicotinamide nucleotide transhydrogenase gene (*Nnt*) [24]. *Nnt* encodes a mitochondrial inner membrane protein that catalyses the reversible reduction of NADP+ by NADH. Mutations in *Nnt* have been associated with aberrant insulin secretion by β pancreatic cells [25]. To prevent any misinterpretation of metabolic data due to the *Nnt* mutation, we transferred the *Alms1* gene trap mutation onto the C57BL6/Ei background which has a normal copy of *Nnt*. *B6Ei.129S2-Alms1^Gt(XH152)Byg/Pjn^* (*Alms1^GT/GT^*) mutant male mice gain less weight but display higher insulin levels compared to the *Alms1* genetrap on a mixed background. As shown in Figure 1a, from 12 weeks of age, *Alms1^GT/GT^* males (n = 15) gained more weight than wt controls (weight at 20 weeks age: 46.2±0.8 vs. 32.2±1.3 g, p<0.001) and had significantly higher blood glucose levels only at 20 weeks of age (233±3.8 vs. 175±1.9 mg/dL p<0.001, Figure 1b). Hyperinsulinemia manifested as early as 8 weeks of age and became dramatically high, reaching the maximum level at 20 weeks (138.3±16.5 vs. 3.2±0.1 ng/ml p<0.001, Figure 1c). Additional clinical comparisons of *Alms1^GT/GT^* and littermates at 6 and 18–21 weeks of age are shown in Table S2. In general, *Alms1^GT/GT^* males have markedly higher levels of cholesterol compared to littermate controls. Six week old *Alms1^GT/GT^* mice had slightly higher leptin levels than controls however values were in the normal range (Table S2, Figure 2e). ALT levels were elevated only in the obese animals indicating liver dysfunction occurs secondary to the metabolic disturbances.

#### Alms1^GT/GT^ AT exhibit adipocyte expansion without macrophage infiltration prior to body weight gain and hyperinsulinemia

To elucidate a possible role of ALMS1 in the control of fat deposition, we analyzed the adipose tissue depots of *Alms1^GT/GT^* at 6 weeks of age, prior to weight gain and the onset of circulating hyperinsulinemia. The weights of SAT and VAT from *Alms1^GT/GT^* mice (n = 15) were significantly higher than those of wt mice (SAT: 0.45±0.03 g *vs*. 0.30±0.01 g; VAT: 0.35±0.03 *vs*. 0.22±0.01 p<0.001, Figure 2a), although body weight did not differ (Table S2). In both *Alms1^GT/GT^* and controls, adipocytes from VAT were larger than those of SAT. However, adipocytes of SAT and VAT of *Alms1^GT/GT^* mice were both larger than the corresponding wt adipocytes (Figure 2b and 2c).

Since adipocyte hypertrophy often predisposes AT to inflammatory infiltration by macrophages, we evaluated *Alms1^GT/GT^* AT by IHC staining for macrophages using anti-F4/80 antibody in both 6 week old (pre-metabolic disease) and 20 week old (overt metabolic disease) animals (Figure S1a). Although VAT and SAT from 20 week old *Alms1^GT/GT^* mice showed distinct clusters of F4/80 positive macrophages, the 6 week old *Alms1^GT/GT^* AT did not show any signs of macrophage infiltration. We quantified by qPCR mRNAs for macrophage markers (*Itgax* = integrin alpha X = CD11c and *Emr1* = EGF-like module containing, mucin–like, hormone receptor-like sequence = F4/80) and inflammatory chemokines (*Ccl3* = chemokine C-C motif ligand 3 = Mip1α and *Ccl2* =  chemokine C-C motif ligand 2 = MCP1) in respect to other well known mouse models of metabolic disease (Lep^ob^ and Lepr^db^). The overall expression of inflammatory genes was very low in AT depots of 6 week old *Alms1^GT/GT^* mice. (Figure S1c). Moreover, the expression of inflammatory genes was similar in *Alms1^GT/GT^* mice (6 weeks) compared to littermate wt controls with the exception of *Itgax* expression in SAT (Figure S1b).

Leptin mRNA (Figure 2d) was upregulated in both adipocyte depots derived from *Alms1^GT/GT^* mice (SAT-2.5 fold increased expression and VAT -4 fold increase), reflecting the increase in the fat depot weight (Figure 2a) and in fat cell size (Figure 2b,c). In addition, plasma leptin (Figure 2e) was slightly elevated in *Alms1^GT/GT^* mice at 6 weeks of age and became significantly higher in *Alms1^GT/GT^* mice at 18–21 weeks of age compared to littermate controls (108.8±62.9 *vs*. 29.5±7.8) (Table S2).

Figure 2f shows the increase in gene expression of several enzymes involved in lipogenic pathways both in *Alms1^GT/GT^* SAT and VAT. *Pck1*, *Fasn*, *Dgat2*, and *Lpl* mRNAs were significantly increased in both fat pad depots, whereas the increased expression of *Dgat1* in *Alms1^GT/GT^* mice was only statistically different in the SAT depot.

*Insulin signaling appears normal in AT of Alms1^GT/GT^ mice.*

**Figure 3:**
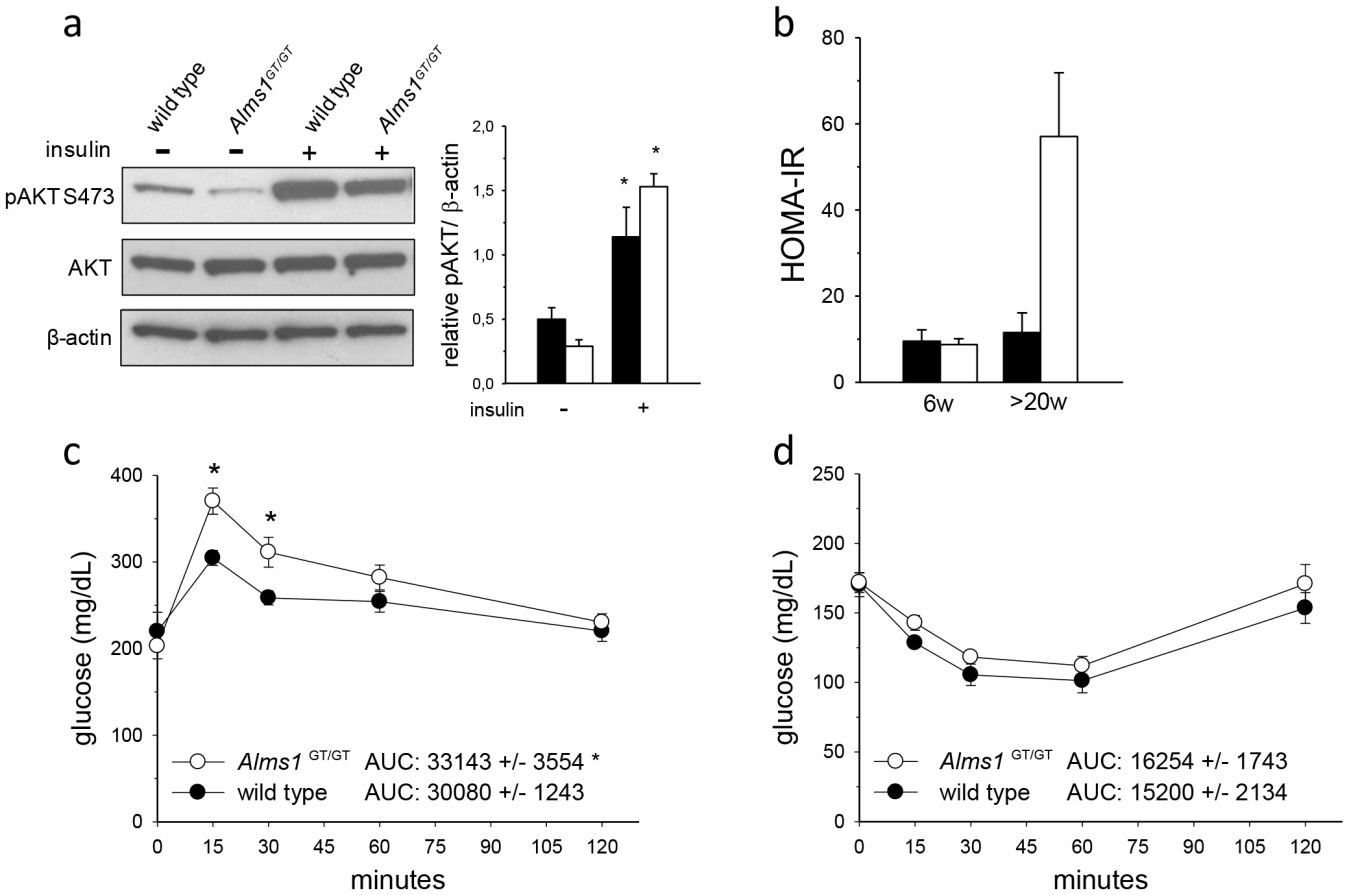
Insulin signaling in adipose tissue and whole body glucose homeostasis of 6 week-old Alms1^GT/GT^ mice. (a) Representative immunoblot and quantification of VAT lysates probed with antibodies: pAKT (S473), AKT, and β-actin from 6 week old wild type (black bars) and *Alms1^GT/GT^* (white bars) mice collected 30 minutes following an intraperitoneal injection of saline (−) or insulin (+). Immunoblots were performed with three independent samples. *p<0.05 wt (−) *vs*. wt (+); *Alms1^GT/GT^* (−) *vs*. *Alms1^GT/GT^* (+). (b) HOMA-IR values of control (black bars) and mutant mice (white bars) at different ages (w = weeks) following a six hour fast (6W, n = 4–5/group;>20W, n = 3/group). Intraperitoneal glucose (c) and insulin (d) tolerance tests were performed at 6 weeks of age in wt (black circle) and *Alms1^GT/GT^* (white circle) mice. Glucose values are means ±SEM; n = 5–7 for each group. The areas under the curve (AUC) are arbitrary units shown as means ±SEM. *p<0.05 wt *vs*. *Alms1^GT/GT^*.

To assess for disturbances in insulin signaling in *Alms1^GT/GT^* mice, we examined the phosphorylation of AKT under basal and insulin stimulated conditions in mice prior to overt metabolic disease at 6 weeks of age. Upon insulin stimulation, pAKT S473 levels increased significantly in both *Alms1* ^GT/GT^ and control VAT compared to basal levels (Figure 3a). There were no significant differences observed in pAKT level between *Alms1^GT/GT^* mice and control littermates indicating insulin signaling occurs via proper AKT activation in VAT of these mice.

#### Alms1^GT/GT^ mutant mice display glucose intolerance in vivo

To examine whole body glucose tolerance and insulin sensitivity in ALMS, we calculated the HOMA-IR index (Figure 3b) and performed intraperitoneal injections of glucose and insulin in fasted 6 week old *Alms1^GT/GT^* mice and control littermates (Figure 3c,d). HOMA-IR values were comparable between mutant and control littermates at 6 weeks of age (9.6±2.6 vs 8.8±1.3, p = 0.79) and increased only in>20 week old *Alms1^GT/GT^* mice. During a glucose tolerance test, *Alms1^GT/GT^* mice had significantly higher glucose levels fifteen minutes post glucose administration compared with their littermate counterparts. However, by 120 minutes post-injection, glucose levels did return close to basal levels in both *Alms1^GT/GT^* and control mice. When challenged with insulin, circulatory glucose decreased in both *Alms1^GT/GT^* and control mice, although levels were slightly (but not significantly) higher in *Alms1^GT/GT^*. The calculated differences of the areas under the curve (AUCs) were significant only in the GTT test (Figure 3c).

#### Total SLC2A4 (GLUT4) content is reduced and its distribution is altered in adipose tissue of 6 week old Alms1^GT/GT^ mice before and after insulin stimulation

**Figure 4:**
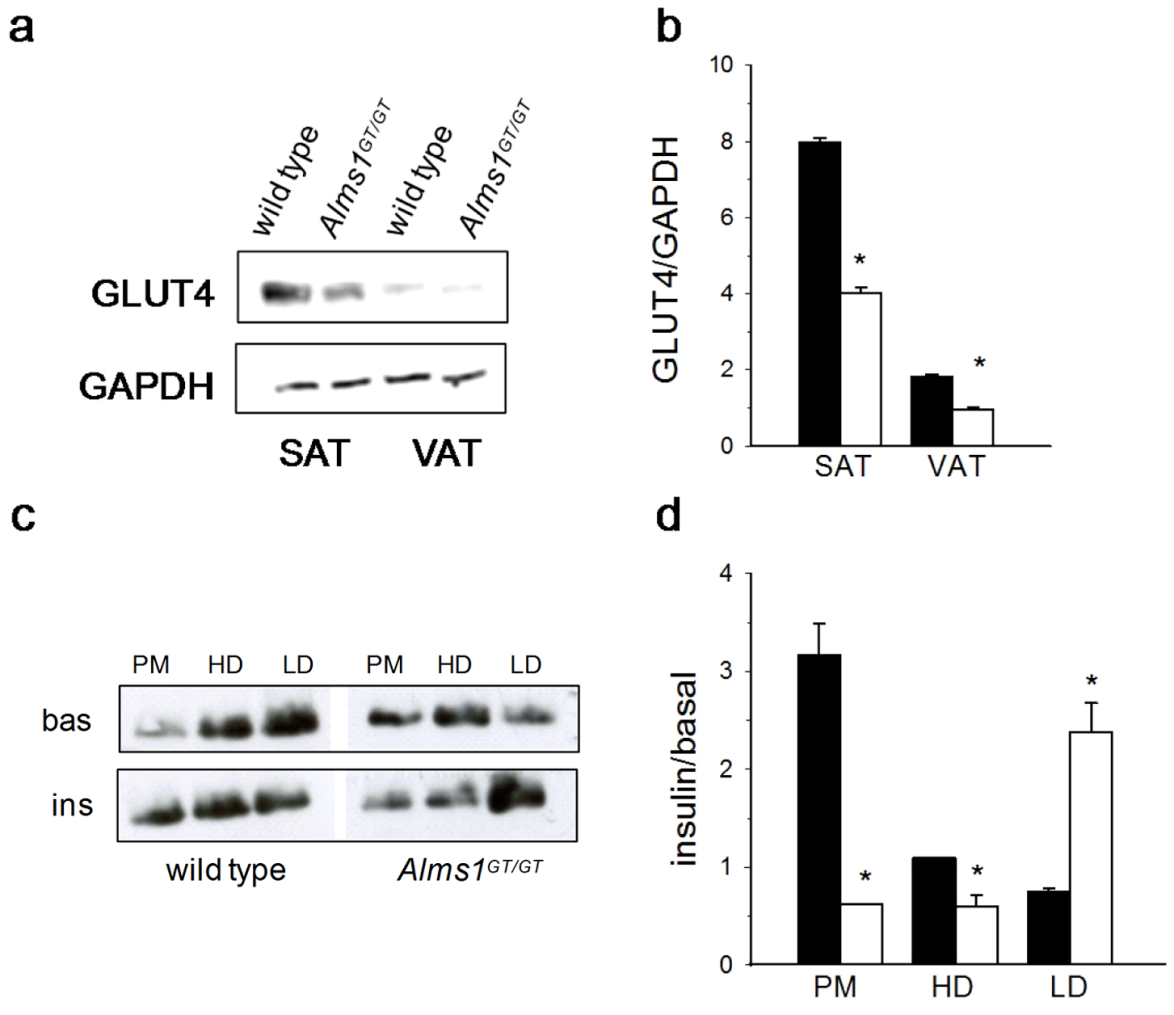
GLUT4 content and subcellular distribution in adipose tissue depots of 6 week-old Alms1^GT/GT^ mice before and after insulin stimulation. (a) Representative western blot of total GLUT4 and (b) quantification (n = 6) normalized for glyceraldehyde-3-phosphate dehydrogenase (GAPDH) content, in subcutaneous (SAT) and visceral (VAT) adipose tissues of wt (black bars) and *Alms1^GT/GT^* (white bars). (c) Representative western blot of GLUT4 distribution in the subcellular compartments (PM = plasma membrane; HD =  high density microsome; LD = low density microsome) in basal conditions (bas) and after insulin stimulation (ins) in SAT pooled from 3 wt and *Alms1^GT/GT^* mice. (d) The plot represents the fold-increase (insulin/basal) in GLUT4 signal after insulin stimulation in every subcellular fraction from SAT of wt (black bars) and *Alms1^GT/GT^* (white bars) mice as mean values ±SEM of 3 western blot quantification. *p<0.05 wt *vs*. *Alms1^GT/GT^*.

Western analysis showed that VAT possessed less GLUT4 when compared to SAT in both *Alms1^GT/GT^* and wt littermates. Interestingly, the glucose transporter content of *Alms1^GT/GT^* was decreased by 50% in both adipose tissue depots (Figure 4a,b). To examine the possible involvement of ALMS1 in GLUT4 trafficking, mice were treated *in vivo* with or without insulin and GLUT4 distribution along the cellular compartments was measured in SAT obtained from *Alms1^GT/GT^* and wt littermates (Figure 4c,d). The distribution pattern of GLUT4 differed in *Alms1^GT/GT^* and control mice both at the basal level as well as upon insulin stimulation. In unstimulated wt mice, the majority of GLUT4 was retained in the recycling endosome compartment (LD) and only a small amount located in the PM while insulin stimulation exerted a threefold increase of GLUT4 translocation to the PM. In contrast, under basal conditions, *Alms1^GT/GT^* displayed a higher quantity of GLUT4 in PM fraction and a reduced quantity in the internal compartments, mainly in the LD fraction. After insulin stimulation, we did not observe an increase in GLUT4 content in the PM fraction, but a 2.4 fold increase in the LD fraction, suggesting alterations in GLUT4 distribution and in insulin-mediated GLUT4 trafficking. Similar results were obtained in VAT although the values for the PM fraction did not reach significance (Figure S2a,b).

### Studies *in vitro*: adipogenic differentiation and function

#### Pre-adipocytes derived from Alms1^GT/GT^ and littermate controls have similar adipogenic potential

**Figure 5:**
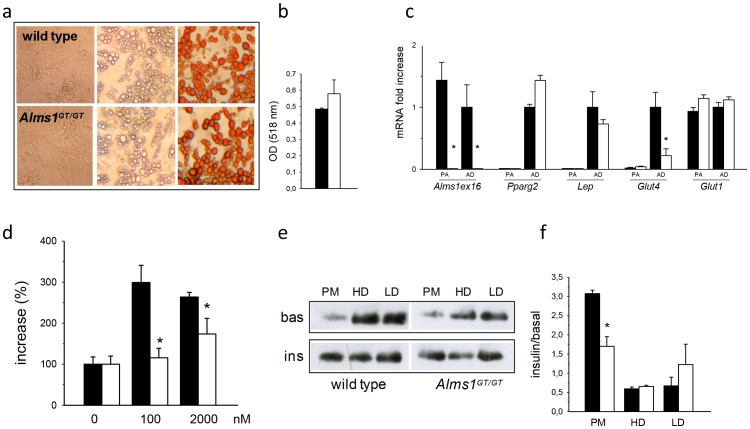
In vitro characterization of pre-adipocyte adipogenic potential and adipocyte insulin responsiveness from 6 week-old Alms1^GT/GT^ mice. (a) Representative pictures of *in vitro* adipogenic differentiation from wt and *Alms1^GT/GT^* SAT pre-adipocytes (32× magnification). Pre-adipocytes (left) were grown in adipogenic medium until fully differentiation (middle) and then stained with Oil Red-O (right). (b) Spectrophotometer ODs from Oil Red O staining of *in vitro* differentiated adipocytes (n = 3) are reported as mean values ±SEM. (c) mRNA expression of reported genes in pre-adipocytes (PA) and mature adipocytes cell cultures (AD) from wt (black bars) and *Alms1^GT/GT^* (white bars) mice was normalized to *Rn18s* content, reported as arbitrary unit mean ratio ±SEM and expressed as fold change with respect to wt AD, arbitrarily set as 1 for each transcript. *p<0.05 wt *vs*. *Alms1^GT/GT^*. (d) Insulin-induced 2DG-uptake of adipocyte cell cultures (n = 3) obtained from wt (black bars) and *Alms1^GT/GT^* (white bars) mice stimulated with different insulin concentration and normalized for total protein content. Data are reported as percent increase (%) over basal uptake (0 nM insulin) which was arbitrarily set as 100 for each group. (e) Representative western blot of GLUT4 distribution in the subcellular compartments (PM = plasma membrane; HD =  high density microsome; LD = low density microsome) in basal conditions (bas) and after insulin stimulation (ins) in adipocyte cultures obtained from SAT of wt and *Alms1^GT/GT^* mice. (f) The plot represents the fold increase (insulin/basal) in GLUT4 signal after insulin stimulation in every subcellular fraction from adipocyte cultures (n = 3) of wt (black bars) and *Alms1^GT/GT^* (white bars) mice as mean values ±SEM. *p<0.05 wt *vs*. *Alms1^GT/GT^*.

We established mouse pre-adipocyte primary cultures from stromal vascular fractions of SAT in *Alms1^GT/GT^* and wt males. The number of cells obtained by collagenase digestion of SAT was comparable between both groups (Figure S3a). We observed that the *in vitro* morphological differentiation and triglyceride accumulation were similar, as shown by Oil Red O staining (Figure 5a) and quantification (Figure 5b). We measured the expression of *Alms1*, *Pparg2*, *Lep*, *Glut4* and *Glut1* in pre-adipocytes (PA) and differentiated adipocytes (AD) of *Alms1^GT/GT^* and controls. Abundant *Alms1* expression was observed only in control cells. *Pparg2*, *Lep* and *Glut4* expression became upregulated upon differentiation of adipocytes in both *Alms1^GT/GT^* and controls, suggesting a similar degree of adipogenic maturation. *Glut1* mRNA levels were similar between pre- and mature adipocytes. *Glut4* mRNA expression was significantly reduced in *Alms1^GT/GT^* mature adipocytes compared to wt (Figure 5c) while *Glut1* levels were similar between the two groups.

#### In vitro analysis of Alms1^GT/GT^ adipocytes reveals defective insulin-dependent glucose uptake and GLUT4 distribution in subcellular compartments

To further examine the underlying mechanism of insulin sensitivity, we evaluated glucose uptake rates in adipocyte cultures *in vitro* from SAT. In pre-adipocytes (n = 3) isolated from SAT of 6 week *Alms1^GT/GT^* and wild type males, insulin stimulated a slight increase in 2-DG uptake, showing the same pattern in both groups (Figure S3b). However, in the *Alms1^GT/GT^* adipocytes differentiated *in vitro* (n = 3), insulin-dependent 2DG-uptake was significantly reduced compared to wt (1.1 *vs*. 3-fold increase at 100 nM and 1.7 *vs*. 2.7 at 2 µM insulin p<0.001, Figure 5d).

We analyzed the GLUT4 distribution to the subcellular compartments in the *in vitro* differentiated mature adipocytes (n = 3) before and after insulin treatment by immunoblot. The GLUT4 distribution in cell culture (Figure 5e and 5f) was similar to that obtained in SAT (Figure 4c,d): under basal conditions, adipocytes from *Alms1^GT/GT^* mice had less GLUT4 in the intracellular compartments (HD and LD) while insulin-induced adipocytes had only 50% of the translocation to the PM compared with wt.

## Discussion

Adipose tissue is a dynamic organ that regulates fat mass and glucose homeostasis often involving the crosstalk with multiple systems (liver, muscles, pancreas and brain). In functionally impaired adipocytes, expansion becomes misregulated and can cause several metabolic dysfunctions, ending in a systemic disease: commonly referred as adiposopathy [2], [26], [27]. In Alström Syndrome, the metabolic alterations manifest early; reduced insulin-stimulated glucose disposal and hyperinsulinemia have been observed in patients as young as 1 year of age and can appear before the start of obesity in children, often evolving to type 2 diabetes during childhood, with variable age of onset [9], [28], [29]. The metabolic impairments are partially manageable by therapeutic interventions [30], [31], but the missing link between ALMS1 disruption and metabolic alterations hampers the identification of a specific treatment.

The present study focused on the effects of *Alms1* disruption on adipose tissue, in a mouse model recapitulating the metabolic disorders observed in ALMS patients. Our results showed that *Alms1^GT/GT^* mice have aberrant insulin signaling either downstream or independent of AKT signaling, before the increase of body weight and circulating insulin levels. Both main adipose tissue depots (SAT and VAT) showed increases in weight and were composed of larger adipocytes, without macrophage infiltration, suggesting an expansion by hypertrophic mechanisms. Adipocyte enlargement has been reported to be associated with inflammatory infiltration and IR, both in animals and humans [32], [33], [34] and pharmacological treatments inducing the formation of small adipocytes (eg. thiazolidinediones) result in increased insulin sensitivity [35]. *Alms1^GT/GT^* mice had increased expression of leptin in SAT and VAT and a slight elevation of plasma leptin levels, although not statistically significant, before the onset of obesity. We previously reported that ALMS patients display elevated blood leptin levels correlating with their body weight [36]; recently we observed (Dr. J. Han, personal communication) that these leptin levels are higher than those of age/weight matched controls, suggesting the presence of leptin resistance.

The elevated gene expression levels of lipogenic enzymes in *Alms1^GT/GT^* AT (both VAT and SAT) could account for the cellular enlargement and weight gain. Importantly, we did not observe any differences in the *in vitro* differentiation process of SAT derived pre-adipocytes obtained from *Alms1^GT/GT^* and wild type mice, suggesting that mutations in *Alms1* do not directly affect adipocyte maturation and that the AT expansion is secondary to multiple *in vivo* interactions. In contrast to our results, Huang-Doran et al. [37] showed that the silencing of *Alms1* causes an impaired adipogenesis in the 3T3-L1 cell line. However, this finding does not explain the obese phenotype present in mouse models and patients with ALMS [9].

We observed a 50% reduction in GLUT4 protein in SAT and VAT of 6 week-old *Alms1^GT/GT^* mice, before the onset of metabolic disease. Interestingly, *Alms1^GT/GT^* AT displayed an altered localization of GLUT4 in the basal state and a reduced translocation of GLUT4 to the PM when insulin-stimulated, both *in vivo* and *in vitro*. Moreover, *in vitro* differentiated adipocytes showed a reduction in insulin-stimulated glucose uptake. Similar findings have been observed in the AS160 knockout (ko) mouse [38], [39]. AS160 is a Rab GTPase activating protein that targets RABs and regulates the intracellular retention of GLUT4 via the formation of specialized GLUT4 storage vesicles (GSVs) [40], [41]. Like *Alms1^GT/GT^* mice, *AS160^-/-^* adipose tissue displays reduced insulin-stimulated glucose uptake, undergoes proper AKT phosphorylation, and shows a decreased basal GLUT4 content and relatively diminished GLUT4 translocation to the PM. Although *AS160^-/-^* males show increased adipocyte expansion in VAT at 6 weeks of age, they do not develop an overt obese phenotype as seen in *Alms1^GT/GT^* mice [38].

In the adipose tissue-specific GLUT4 ko mouse model [7], the absence of GLUT4 resulted in reduced insulin-stimulated glucose transport in adipocytes, but normal fat accumulation. In contrast, our ALMS model displayed enlarged fat pads together with increased expression of lipogenic enzymes at 6 weeks of age, suggesting that AT expansion occurs despite reduced insulin-dependent glucose uptake. *Alms1^GT/GT^* mice develop a more profound hyperinsulinemia than that present in the AT-specific GLUT4 ko mice, which could account for *de novo* lipogenesis by means of the uncoupled mechanism of insulin action on glucose flux and lipid accumulation, described by Gonzalez E. et al. [42]. In this view, *Alms1* disruption generates a novel model which involves the pathological expansion of AT, typical of obesity models [43], [44] with a dramatic hyperinsulinemia, characterizing lipodystrophic models [45], [46] and suggesting that very high insulin levels could overcome a primary AT IR and drive its expansion to obesity.

*Alms1^GT/GT^* mice, prior to metabolic disease, showed marked systemic glucose intolerance, but were able to clear the glucose from circulation within 2 hours of glucose injection. This suggests the existence of a compensatory mechanism, perhaps in other organ systems, for glucose tolerance in *Alms1^GT/GT^* mice. The role of ALMS1 in the regulation of glucose uptake in muscle and brown adipose tissue is yet to be elucidated.

Insulin stimulates increased glucose uptake in AT and muscle by a series of complex signaling events that result in the redistribution of GLUT4 from intracellular vesicles to the cell membrane [8], [47]. GLUT4 translocation may occur via a PI3-dependent mechanism that results in the phosphorylation of AKT which in turn activates downstream substrates such as AS160 and TBC1D1 [48]. Our results show that *Alms1^GT/GT^* mice have the ability to respond to insulin by AKT phosphorylation. This suggests that the impairment of GLUT4 translocation either lies downstream of AKT activation or may be independent of AKT signaling. It remains to be determined whether ALMS may physically interact with AKT downstream substrates to regulate the formation of GSVs or deactivate its retention.

The actin cytoskeleton plays an important role in insulin-stimulated GLUT4 transport [47]. Upon insulin stimulation, cortical actin filaments undergo remodeling, resulting in ruffling of the plasma membrane. Previously, we reported disturbances in actin architecture and transferrin receptor trafficking in fibroblasts derived from ALMS patients [16]. We also showed that ALMS1 interacts with Actinin 4 (ACTN4) and other members of the cytoskeleton-associated recycling or transport (CART) complex [16]. In muscle, ACTN4 has been associated with GLUT4 trafficking and its knockdown impaired the transporter localization to the PM after insulin stimulation [49], [50]. It remains plausible that ALMS may be involved in the movement of GLUT4 vesicles along microtubules or its docking to the PM through its interaction with the actin cytoskeleton.

In conclusion, we showed several defects of GLUT4 in adipose tissue of *Alms1^GT/GT^*: reduction of total protein content, mislocalization, impairment in insulin-induced translocation, suggesting a role for ALMS1 in glucose homeostasis and GLUT4 translocation. All these defects emerge before the development of overt metabolic disease and may provide a possible explanation for the impaired insulin-stimulated glucose uptake and the compensatory hyperinsulinemia. The secondary AT expansion may be due to an increase in *de novo* lipogenesis stimulated by the very high insulin levels while the underlying mechanism responsible for reduced GLUT4 expression remains to be elucidated. Although AT dysfunction certainly is an early consequence of *Alms1* mutations, we cannot exclude the possibility that other systemic aberrations could contribute to the metabolic diseases in *Alms1^GT/GT^* and in ALMS patients, such as the impairment in leptin receptor trafficking resulting in leptin-resistance (similar to what was observed in Bardet Biedl syndrome [51]), the reduction of cilia number in the hypothalamus [52], or the alteration of insulin vesicle release mediated by centrosomal proteins in pancreatic β cells [53]. Ultimately, a better understanding of the crosstalk involved in insulin signaling may lead to improved therapeutic and prevention strategies of ALMS and other metabolic diseases involving IR.

## Supporting Information

Figure S1 **Adipose tissue inflammation in 6 week-old Alms1^GT/GT^ mice.** (a) A representative DAB immunostaining with anti-macrophage marker F4/80 antibody in SAT and VAT of 6 and 20 week old *Alms1^GT/GT^* mice. Scale bar  = 100 µm. Arrows depict areas of macrophage infiltration. (b) mRNA expression of several inflammatory genes in SAT and VAT from 3 wt (black bars) and 3 *Alms1^GT/GT^* (white bars) mice [*Itgax*: integrin alpha X (CD11c), *Emr1*: EGF-like module containing, mucin-like, hormone receptor-like sequence 1(F4/80), *Ccl3*: chemokine (C-C motif) ligand 3 (Mip 1α), *Ccl2*: chemokine (C-C motif) ligand 2 (MCP1)]. Each transcript was normalized to *Rn18s* content; results are reported as arbitrary unit mean ratio ±SEM and are expressed as fold change with respect to wt, arbitrarily set as 1 for each transcript. *p<0.05 wt *vs*. *Alms1^GT/GT^*. (c) *Itgax*, *Emr1*, *Ccl3*, and *Ccl2* mRNA expression in SAT (black bars) and VAT (white bars) of 10 week old *Lep^ob^* and *Lepr^db^* heterozygous controls (ob/+; db/+) and homozygous mice (ob/ob; db/db) compared to 6 week old *Alms1^GT/GT^* and littermate wt controls. Data are normalized to *Rn18s* (*18S*) content and reported as arbitrary unit mean ratio ±SEM. *p<0.05 wt *vs*. *Alms1^GT/GT^*; ob/+ *vs* ob/ob; db/+ *vs* db/db.
(TIF)

Figure S2 **GLUT4 subcellular distribution in visceral adipose tissue of 6 week-old Alms1^GT/GT^ mice before and after insulin stimulation.** (a) Representative western blot of GLUT4 distribution in the subcellular compartments (PM = plasma membrane; HD =  high density microsome; LD = low density microsome) in basal conditions (bas) and after insulin stimulation (ins) in VAT pooled from 3 wt and *Alms1^GT/GT^* mice. (b) The plot represents the fold-increase (insulin/basal) in GLUT4 signal after insulin stimulation in every subcellular fraction from VAT of wt (black bars) and *Alms1^GT/GT^* (white bars) mice as mean values ±SEM of 3 western blot quantification. *p<0.05 wt *vs*. *Alms1^GT/GT^*.
(TIF)

Figure S3 **Pre-adipocyte characterization in 6 week-old Alms1^GT/GT^ mice.** (a) The pre-adipocyte number has been quantified by cell counting in subcutaneous adipose tissue (SAT) of 3 wt (black bars) and 3 *Alms1^GT/GT^* (white bars) mice. Data are reported as mean value (×10^6^) ±SEM and normalized to the SAT depot weight (×10^6^/g) (b) Insulin-induced 2DG-uptake of pre-adipocyte cell cultures (n = 3) obtained from SAT of wt (black bars) and *Alms1^GT/GT^* (white bars) mice stimulated with different insulin concentration and normalized for total protein content. Data are reported as percent increase (%) over basal uptake (0 nM insulin) which was arbitrarily set as 100 for each group.
(TIF)

Table S1 **qPCR conditions.** Primer sequences and concentrations, cycling parameters and amplicon size were reported for each mRNA quantified by real-time PCR. *Lep* =  leptin; *Pck1* = Phosphoenolpyruvate carboxykinase 1, cytosolic; *Fasn* = fatty acid synthase; *Dgat1* = diacylglycerol O-acyltransferase 1; *Dgat2* =  diacylglycerol O-acyltransferase *2*; *Lpl* =  lipoprotein lipase; *Itgax* =  integrin alpha X (CD11c); *Emr1* = EGF-like module containing, mucin-like, hormone receptor-like sequence 1(F4/80); *Ccl3* =  chemokine (C-C motif) ligand 3 (Mip 1α); *Ccl2* =  chemokine (C-C motif) ligand 2 (MCP1), *Alms1* =  Alström Syndrome 1; *Pparg* =  peroxisome proliferator-activated receptor gamma; *Slc2a4* = solute carrier family 2 (facilitated glucose transporter), member 4 (Glut4); *Slc2a1* =  solute carrier family 2 (facilitated glucose transporter), member 1 (Glut1); *Rn18s* = 18S ribosomal RNA.
(DOC)

Table S2 **Plasma chemistries in non-fasted Alms1^GT/GT^ mice and controls before and after the onset of obesity.** Values are expressed as means ±SEM. An asterisk (*) indicates a statistically significant difference between mutant and control mice at the age indicated (P<0.05, two tailed t-test). Littermate controls are *Alms1^+/GT^* or *Alms1^+/+^*. Number of animals in each group: 8–15 (6W) and 6–15 (18–21W). † LDL levels, n = 4–6 per group.
(DOCX)
