# Supplementary figures and images for: GLUT4 Defects in Adipose Tissue Are Early Signs of Metabolic Alterations in Alms1GT/GT, a Mouse Model for Obesity and Insulin Resistance
Source: PLoS One. 2014 Oct 9;9(10):e109540. doi: 10.1371/journal.pone.0109540 (PMC4192353; doi:10.1371/journal.pone.0109540)

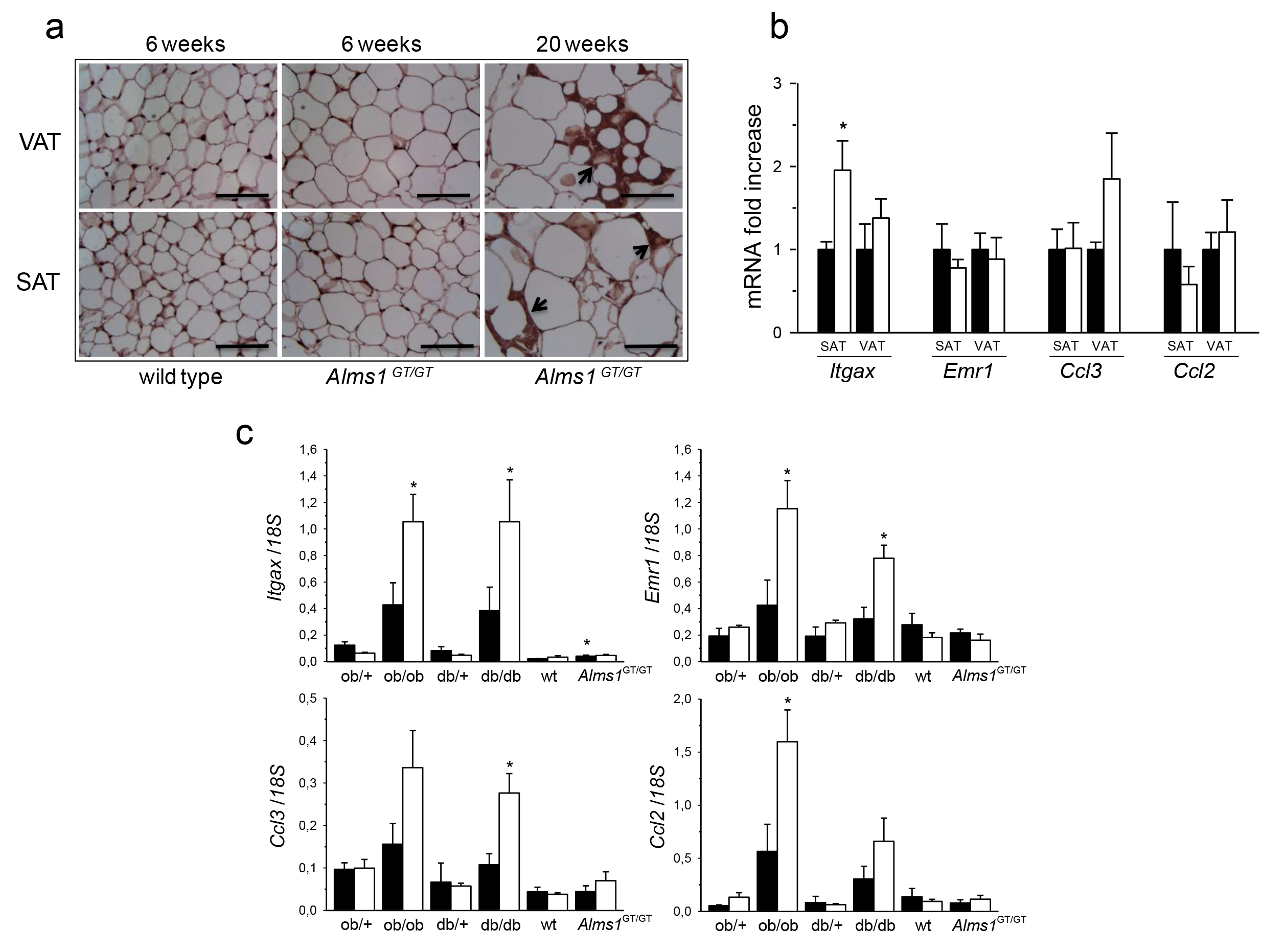

Supplement: Figure S1 — Adipose tissue inflammation in 6 week-old Alms1GT/GT mice. (a) A representative DAB immunostaining with anti-macrophage marker F4/80 antibody in SAT and VAT of 6 and 20 week old Alms1GT/GT mice. Scale bar = 100 µm. Arrows depict areas of macrophage infiltration. (b) mRNA expression of several inflammatory genes in SAT and VAT from 3 wt (black bars) and 3 Alms1GT/GT (white bars) mice [Itgax: integrin alpha X (CD11c), Emr1: EGF-like module containing, mucin-like, hormone receptor-like sequence 1(F4/80), Ccl3: chemokine (C-C motif) ligand 3 (Mip 1α), Ccl2: chemokine (C-C motif) ligand 2 (MCP1)]. Each transcript was normalized to Rn18s content; results are reported as arbitrary unit mean ratio ±SEM and are expressed as fold change with respect to wt, arbitrarily set as 1 for each transcript. *p<0.05 wt vs. Alms1GT/GT. (c) Itgax, Emr1, Ccl3, and Ccl2 mRNA expression in SAT (black bars) and VAT (white bars) of 10 week old Lepob and Leprdb heterozygous controls (ob/+; db/+) and homozygous mice (ob/ob; db/db) compared to 6 week old Alms1GT/GT and littermate wt controls. Data are normalized to Rn18s (18S) content and reported as arbitrary unit mean ratio ±SEM. *p<0.05 wt vs. Alms1GT/GT; ob/+ vs ob/ob; db/+ vs db/db. (TIF) [file pone.0109540.s001.tif]

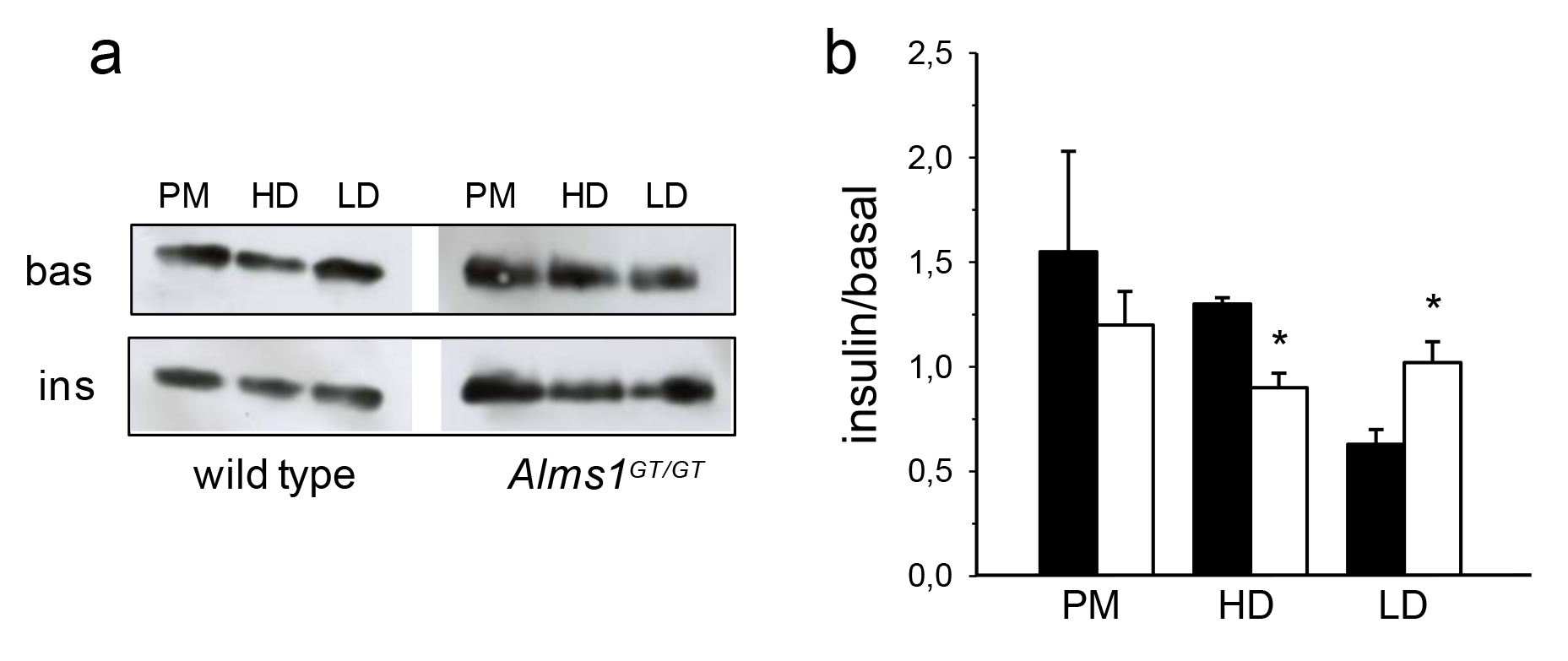

Supplement: Figure S2 — GLUT4 subcellular distribution in visceral adipose tissue of 6 week-old Alms1GT/GT mice before and after insulin stimulation. (a) Representative western blot of GLUT4 distribution in the subcellular compartments (PM = plasma membrane; HD = high density microsome; LD = low density microsome) in basal conditions (bas) and after insulin stimulation (ins) in VAT pooled from 3 wt and Alms1GT/GT mice. (b) The plot represents the fold-increase (insulin/basal) in GLUT4 signal after insulin stimulation in every subcellular fraction from VAT of wt (black bars) and Alms1GT/GT (white bars) mice as mean values ±SEM of 3 western blot quantification. *p<0.05 wt vs. Alms1GT/GT. (TIF) [file pone.0109540.s002.tif]

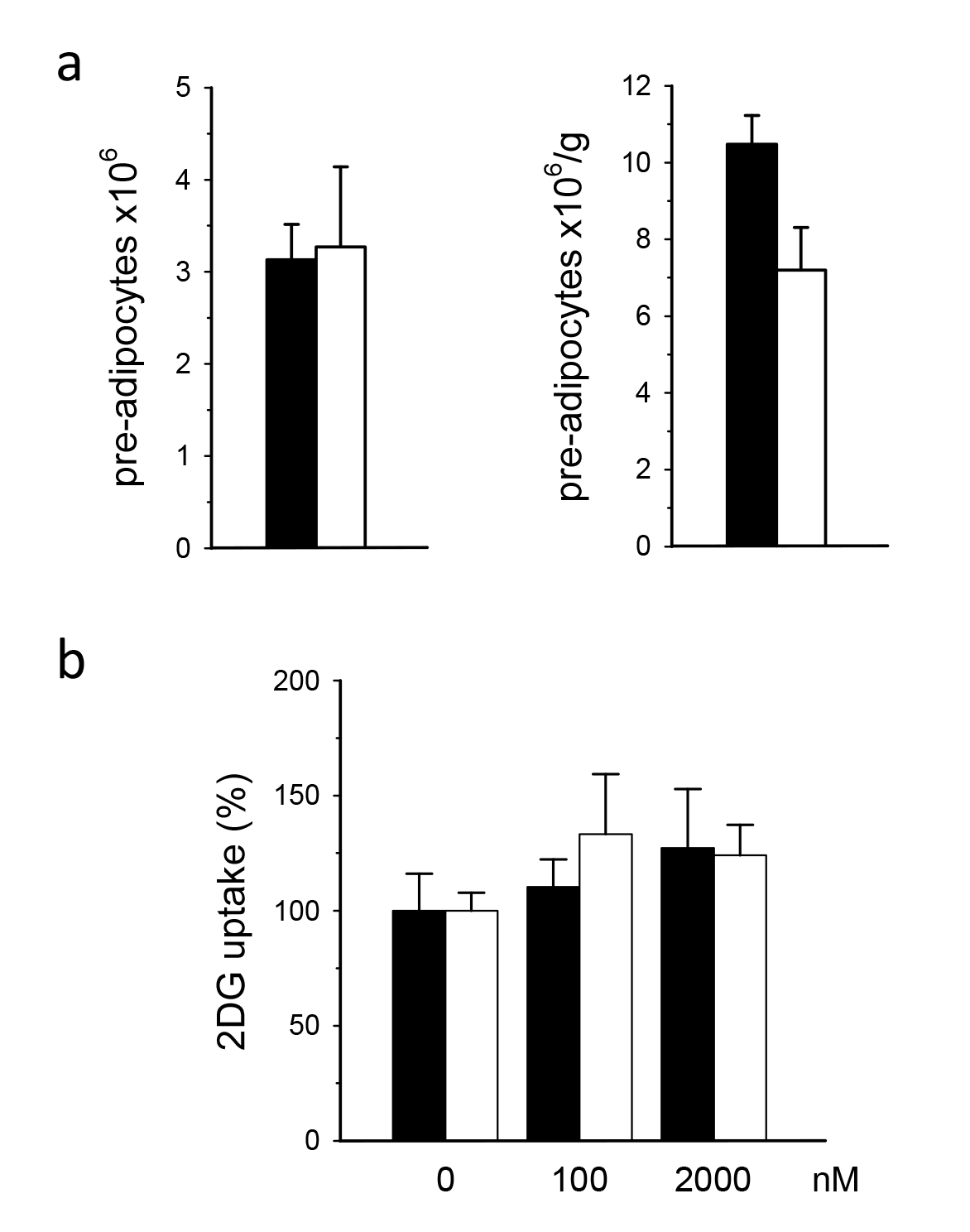

Supplement: Figure S3 — Pre-adipocyte characterization in 6 week-old Alms1GT/GT mice. (a) The pre-adipocyte number has been quantified by cell counting in subcutaneous adipose tissue (SAT) of 3 wt (black bars) and 3 Alms1GT/GT (white bars) mice. Data are reported as mean value (×106) ±SEM and normalized to the SAT depot weight (×106/g) (b) Insulin-induced 2DG-uptake of pre-adipocyte cell cultures (n = 3) obtained from SAT of wt (black bars) and Alms1GT/GT (white bars) mice stimulated with different insulin concentration and normalized for total protein content. Data are reported as percent increase (%) over basal uptake (0 nM insulin) which was arbitrarily set as 100 for each group. (TIF) [file pone.0109540.s003.tif]
