# Supplementary material for: GLUT4 Defects in Adipose Tissue Are Early Signs of Metabolic Alterations in Alms1GT/GT, a Mouse Model for Obesity and Insulin Resistance
Source: PLoS One. 2014 Oct 9;9(10):e109540. doi: 10.1371/journal.pone.0109540 (PMC4192353; doi:10.1371/journal.pone.0109540)
Supplement: Table S1 — qPCR conditions. Primer sequences and concentrations, cycling parameters and amplicon size were reported for each mRNA quantified by real-time PCR. Lep = leptin; Pck1 = Phosphoenolpyruvate carboxykinase 1, cytosolic; Fasn = fatty acid synthase; Dgat1 = diacylglycerol O-acyltransferase 1; Dgat2 = diacylglycerol O-acyltransferase 2; Lpl = lipoprotein lipase; Itgax = integrin alpha X (CD11c); Emr1 = EGF-like module containing, mucin-like, hormone receptor-like sequence 1(F4/80); Ccl3 = chemokine (C-C motif) ligand 3 (Mip 1α); Ccl2 = chemokine (C-C motif) ligand 2 (MCP1), Alms1 = Alström Syndrome 1; Pparg = peroxisome proliferator-activated receptor gamma; Slc2a4 = solute carrier family 2 (facilitated glucose transporter), member 4 (Glut4); Slc2a1 = solute carrier family 2 (facilitated glucose transporter), member 1 (Glut1); Rn18s = 18S ribosomal RNA. (DOC) [file pone.0109540.s004.doc]

| **GENE**  **(Official Symbol)** | **FORWARD**  **(5’-3’)** | **REVERSE**  **(5’-3’)** | **PRIMER (F/R)** | **TERMAL**  **CONDITIONS** | **AMPLICON SIZE (bp)** |
| --- | --- | --- | --- | --- | --- |
| ***Lep*** | TCCAGAAAGTCCAGGATGACAC | CACATTTTGGGAAGGCAGG | 300nM/  300nM | 95°Cx15’’  60°Cx1’  40cycles | 211 |
| ***Pck1*** | CGATGACATTGCCTGGATGAA | TGGATGGTTTTAATGGCATTTGG | 300nM/  300nM | 95°Cx15’’  60°Cx1’  40cycles | 129 |
| ***Fasn*** | CACATCCTAGGCATCCGAGA | CAGATCGTGTTCTCGTTCCA | 300nM/  300nM | 95°Cx15’’  60°Cx1’  40cycles | 120 |
| ***Dgat1*** | AGGTGCCATCGTCTGCAAGATT | TCCACCAGGATGCCATACTTGATA | 300nM/  300nM | 95°Cx15’’  60°Cx1’  40cycles | 149 |
| ***Dgat2*** | TGGCTGGCATTTGACTGGAA | TTCACCAGCTGGATGGGAAAGT | 300nM/  300nM | 95°Cx15’’  60°Cx1’  40cycles | 113 |
| ***Lpl*** | TCAGAGCCAAGAGAAGCAGCAA | TGTGTTGCTTGCCATCCTCA | 300nM/  300nM | 95°Cx15’’  60°Cx1’  40cycles | 117 |
| ***Itgax*** | CTGGATAGCCTTTCTTCTGCTG | GCACACTGTGTCCGAACTCA | 300nM/  300nM | 95°Cx15’’  60°Cx1’  40cycles | 113 |
| ***Emr1*** | CTGGGATCCTACAGCTGCTC | AGGAGCCTGGTACATTGGTG | 300nM/  300nM | 95°Cx15’’  60°Cx1’  40cycles | 301 |
| ***Ccl3*** | TTCTCTGTACCATGACACTCTGC | CGTGGAATCTTCCGGCTGTAG | 300nM/  300nM | 95°Cx15’’  60°Cx1’  40cycles | 100 |
| ***Ccl2*** | CCCAATGAGTAGGCTGGAGA | TCTGGACCCATTCCTTCTTG | 300nM/  300nM | 95°Cx15’’  55°Cx1’  40cycles | 125 |
| ***Alms1 ex16*** | GTGGTGCCTGTGATACGAAAG | AGGCCCGGAGTGAAT | 300nM/  300nM | 95°Cx15’’  60°Cx1’  40cycles | 95 |
| ***Pparg (isoform 2)*** | TTCGCTGATGCACTGCCTATGA | GAATGCGAGTGGTCTTCCATCA | 300nM/  300nM | 95°Cx15’’  60°Cx1’  40cycles | 128 |
| ***Slc2a4*** | TGTCGCTGGTTTCTCCAACTG | CCATACGATCCGCAACATACTG | 300nM/  300nM | 95°Cx15’’  60°Cx1’  40cycles | 73 |
| ***Slc2a1*** | ATCCCATCCACCACATCA | AAGCACAGCAGCCACAAAGG | 300nM/  300nM | 95°Cx15’’  60°Cx1’  40cycles | 162 |
| ***Rn18s*** | CGGCTACCACATCCAAGGAA | GCTGGAATTACCGCGGCT | 100nM/  100nM | 95°Cx15’’  60°Cx1’  35 cycles | 186 |

**Table S1.** *qPCR conditions*. Primer sequences and concentrations, cycling parameters and amplicon size were reported for each mRNA quantified by real-time PCR. *Lep=* leptin; *Pck1=*Phosphoenolpyruvate carboxykinase 1, cytosolic*; Fasn=*fatty acid synthase*; Dgat1=*diacylglycerol O-acyltransferase 1*; Dgat2=* diacylglycerol O-acyltransferase *2; Lpl=* lipoprotein lipase*; Itgax*= integrin alpha X (CD11c); *Emr1*= EGF-like module containing, mucin-like, hormone receptor-like sequence 1(F4/80); *Ccl3*= chemokine (C-C motif) ligand 3 (Mip 1α); *Ccl2*= chemokine (C-C motif) ligand 2 (MCP1), *Alms1=* Alström Syndrome 1; *Pparg=* peroxisome proliferator-activated receptor gamma; *Slc2a4=*solute carrier family 2 (facilitated glucose transporter), member 4 (Glut4)*; Slc2a1=* solute carrier family 2 (facilitated glucose transporter), member 1 (Glut1)*; Rn18s=*18S ribosomal RNA.
