# Supplementary material for: GLUT4 Defects in Adipose Tissue Are Early Signs of Metabolic Alterations in Alms1GT/GT, a Mouse Model for Obesity and Insulin Resistance
Source: PLoS One. 2014 Oct 9;9(10):e109540. doi: 10.1371/journal.pone.0109540 (PMC4192353; doi:10.1371/journal.pone.0109540)
Supplement: Table S2 — Plasma chemistries in non-fasted Alms1GT/GT mice and controls before and after the onset of obesity. Values are expressed as means ±SEM. An asterisk (*) indicates a statistically significant difference between mutant and control mice at the age indicated (P<0.05, two tailed t-test). Littermate controls are Alms1+/GT or Alms1+/+. Number of animals in each group: 8–15 (6W) and 6–15 (18–21W). † LDL levels, n = 4–6 per group. (DOCX) [file pone.0109540.s005.docx]

**Table S2.**

|  | 6W | | 18-21W | |
| --- | --- | --- | --- | --- |
| Clinical Phenotype | *Alms1^GT/GT^* | Littermate  control | *Alms1^GT/GT^* | Littermate control |
| Body weight (g) | 19.7 ± 0.4 | 19.2 ± 0.5 | 46.2 ± 2.1* | 32.2 ± 1.3 |
| Glucose (mg/dl) | 200 ± 9 | 179 ± 8 | 233 ± 4* | 175 ± 2 |
| Insulin (ng/ml) | 1.4 ± 0.2 | 1.0 ± 0.2 | 134 ± 24* | 3.2 ± 0.3 |
| Leptin (ng/ml) | 4.3 ± 1.3 | 2.8 ± 1.6 | 108.8 ± 62.9* | 29.5 ± 7.8 |
| Triglycerides (mg/dl) | 127 ± 14 | 107 ±11 | 94 ± 22 | 131 ± 17 |
| Free Fatty Acids (mEq/L) | 1.3 ± 0.10 | 1.2 ± 0.26 | 1.0 ± 0.12 | 1.3 ± 0.13 |
| Total Cholesterol (mg/dl) | 109 ± 3* | 97 ± 3 | 140± 6* | 94 ± 4 |
| HDL (mg/dl) | 96 ± 8* | 84 ± 8 | 115 ± 7* | 80 ± 7 |
| LDL (mg/dl)† | 8.7 ± 2.3 | 7.8 ± 1.8 | 7.5 ± 1.2 | 4.5 ± 1.0 |
| ALT (IU/L) | 31 ± 3 | 33 ± 4 | 146 ± 41 | 49 ± 4 |

**Plasma chemistries in non-fasted Alms1^GT/GT^ mice and controls before and after the onset of obesity.** Values are expressed as means ± SEM. An asterisk (*) indicates a statistically significant difference between mutant and control mice at the age indicated (P<0.05, two tailed t-test). Littermate controls are *Alms1^+/GT^* or *Alms1^+/+^*. Number of animals in each group: 8-15 (6W) and 6-15 (18-21W). † LDL levels, n=4-6 per group.
